# Affect Labeling and Reappraisal as an Emotion Regulation Strategy

**DOI:** 10.1007/s42761-026-00362-z

**Published:** 2026-03-30

**Authors:** Yael Ariely, Aviv Mokady, Niv Reggev, Gideon E. Anholt

**Affiliations:** 1 Faculty of Humanities and Social Science, Department of Psychology, Ben-Gurion University of the Negev, Beer Sheva, Israel; 2 School of Brain Sciences and Cognition, Ben-Gurion University of the Negev, Beer Sheva, Israel

**Keywords:** Affect labeling, Emotion regulation, Reappraisal, Emotion naming

## Abstract

Affect labeling, the process of identifying and verbally expressing emotions, is being increasingly studied as a naturally occurring emotional process with regulatory consequences. Despite the growing interest, the mechanisms underlying affect labeling remain unclear, with studies yielding contradictory results. While some research supports its positive impact on emotional regulation, a study by Nook et al. (2021) found that affect labeling hindered subsequent reappraisal, highlighting the need for further investigation. This study replicates and extends Nook et al. (2021), hypothesizing that the positive effects of affect labeling on reappraisal might emerge over time. In two studies (total *N* = 226), participants were randomly assigned to four conditions, varying in their use of affect labeling and reappraisal while viewing 24 negatively valenced images. Participants either passively observed, labeled affect, reappraised, or combined labeling and reappraisal. Participants rated their emotional responses immediately and again one to two days later. Results of both studies mirrored those of Nook et al. (2021), with affect labeling reducing the effectiveness of reappraisal. However, follow-up measurements did not support the hypothesis of delayed positive effects, instead showing a return to baseline measures. These findings suggest that the effects of affect labeling and reappraisal diminish over time. The study corroborates Nook et al. (2021), indicating that affect labeling may solidify emerging emotions and limit emotional regulation flexibility.

**Supplementary Information:**

The online version contains supplementary material available at 10.1007/s42761-026-00362-z.

Affect labeling, i.e., the process of identifying and verbalizing one’s current emotional experience, has been increasingly studied as an important emotion regulation process within affective science. Theoretical models rooted in the constructionist view of emotion suggest that labeling affects plays a central role in shaping the way individuals experience and regulate emotions (Barrett, 2006, 2017; Lindquist et al., 2015). By transforming undifferentiated affect into discrete emotional categories through language, affect labeling may influence not only emotional clarity and the capacity for adaptive self-regulation, but also attenuate negative emotional responses. As such, it constitutes a fundamental component of emotional functioning, operating across a range of everyday situations, whether in private reflection, social interactions, or structured emotional tasks (Torre & Lieberman, 2018).

Indeed, numerous studies provide evidence supporting the notion that affect labeling has a dampening effect on negative emotional responses. For instance, research has consistently shown that expressing emotions, whether verbally or in writing, can significantly reduce distress and contribute to better mental and physical health compared to situations where emotions are not expressed at all (Frattaroli, 2006; Pennebaker, 1997; Pennebaker & Beall, 1986). Furthermore, affect labeling was found to be as effective in reducing self-reported distress as other emotion regulation strategies, i.e., reappraisal and distraction (Lieberman et al., 2011). Extending these findings to real-world contexts, Fan et al. (2019) analyzed the emotional trajectories of Twitter users and found that explicit emotion labeling was followed by a rapid decline in negative affect, indicating affect labeling’s efficacy in naturalistic settings.

Further support for the regulatory role of affect labeling can be found in a line of research regarding alexithymia, a personality trait that involves deficiencies in the ability to recognize, identify and describe one’s own emotions. Individuals with high levels of alexithymia tend to use reappraisal less frequently as an emotion regulation strategy (Swart et al., 2009). Moreover, they are more prone to suffer from mental health difficulties, especially depressive disorders (Leweke et al., 2011) and psychosomatic diseases (Sifneos, 1973), compared with subjects who are low with alexithymia traits. These findings suggest that the capacity to label emotions may be a prerequisite for the flexibly deployment of emotion regulation strategies.

Finally, neuroimaging results suggest that affect labeling causes inhibition in limbic regions in the brain, specifically the amygdala, a part of the brain associated with fear and anxiety (Lieberman et al., 2007; Yoshimura et al., 2023). Moreover, this inhibition was found to be inversely correlated with increased activity in the right ventrolateral prefrontal cortex, a connectivity pathway consistently associated with successful emotion regulation, as indicated in a recent meta-analysis (Berboth & Morawetz, 2021). Furthermore, affect labeling increased the coupling between the amygdala and the ventrolateral prefrontal regions (Yoshimura et al., 2023). These findings suggest that language-based processes can modulate neural responses to affective stimuli, supporting the role of affect labeling in influencing emotional reactivity through top-down mechanisms.

Despite growing interest, the mechanisms by which affect labeling contributes to emotion regulation remain unclear. While many studies emphasize its regulatory benefits, other research points to more complex associations. For example, Vine et al. (2020) found that individuals with a broader negative emotion vocabulary reported higher levels of psychological distress, and DeLap et al. (2024) documented similar patterns in adolescents. These findings suggest that although the capacity for emotion labeling may support regulation, it may also, in some cases, reflect or exacerbate underlying emotional difficulties—highlighting the importance of contextual and individual factors in understanding the effects of affect labeling. In this context, affect labeling has been conceptualized as an emotion regulation strategy that operates incidentally unlike more deliberate strategies such as reappraisal or distraction (Lieberman et al., 2011). This distinction has informed hypotheses about affect labeling’s potential role in facilitating other regulatory strategies.

One such hypothesis proposes that labeling potentiates the use of another important regulation strategy, specifically, cognitive reappraisal (Moyal et al., 2014). Reappraisal refers to a cognitive shift in the way one interprets a stimulating and emotional event, in order to reduce the negative affect it elicits and change associated response tendencies (Gross, 1998). According to the process model of emotion regulation, reappraisal operates at a later stage in the emotion-generative process, following initial appraisal of the stimulus (Thiruchselvam et al., 2011). In this context, affect labeling may serve to enhance the clarity of emotional experiences, thereby providing a more defined target for subsequent reappraisal efforts. This notion aligns with Denham and Burton’s (2003) assertion that recognizing and labeling emotions is a critical step in developing the ability to regulate emotional expressions in socially acceptable ways.

Accordingly, Moyal et al. (2014) suggested that labeling emotions intentionally serves as an appraisal and may improve the efficiency of reappraisal. This hypothesis is supported by findings from research on alexithymia reviewed above, which demonstrate that alexithymic individuals are less prone to use reappraisal compared to individuals with low alexithymic traits (Swart et al., 2009). Further support for the potential facilitative role of affective labeling to cognitive reappraisal is suggested by a study in which participants induced with negative emotion by listening to aversive emotional auditory scripts. The study demonstrated that participants with borderline personality disorder who used affect labeling prior to cognitive reappraisal or mindfulness experienced less distress than those who did not use affect labeling, though the same effect was not found in healthy participants, for whom the levels of distress stayed the same with or without affect labeling (Fitzpatrick et al., 2019).

Finally, a study by Vine et al. (2019) showed that the level of detail in emotion labeling may influence individuals’ selection of emotion regulation strategies, so that using fewer words for labeling an emotion (i.e., categorical labeling) generates more problem solving and reappraisal strategies. This study provides further support to Moyal et. al.‘s (2014) hypothesis that affect labeling potentiates the use of reappraisal, though the participants merely stated their tendencies rather than engaged in any regulating strategies, thus, leaving the effect of affect labeling on reappraisal yet uncertain.

Despite the evidence presented here, a study conducted by Nook et al. (2021) presented with findings that contradict the hypothesis that affect labeling potentiates reappraisal by demonstrating that affect labeling inhibited emotion regulation. Specifically, results showed that labeling alone had not affected the level of distress compared to no labeling, and that labeling impeded the effectiveness of subsequent use of reappraisal when compared to reappraisal without preceding affect labeling (Nook et al., 2021). The researchers hypothesized that one reason contributing to the impediment of effectiveness is the “crystalizing” effect of emotion naming, solidifying initial appraisals associated with the chosen emotion word and limiting the generation of alternative appraisals. Additionally, they suggest that the cognitive effort required for emotion naming could diminish motivation for engaging in subsequent regulation efforts. Furthermore, they highlight the significance of both the manner and timing of emotion naming in influencing regulation outcomes. For instance, more elaborate emotion naming has been linked to less optimal regulatory strategies, while the timing of naming may impact short-term crystallization of affect versus long-term facilitation of self-regulation (Vine et al., 2019).

The current studies aim to test a hypothesis that may account for the unexpected detrimental effects of affect labeling on subsequent cognitive change. Specifically, these effects might not reflect a generalizable phenomenon but rather stem from the constraints of a time-limited experimental design. According to Gross’s process model of emotion regulation (1998), regulation strategies involving emotion engagement (such as affect labeling) may take longer to manifest their regulatory impact than regulation strategies that involve disengaging with the emotion (Thiruchselvam et al., 2011). A similar pattern has been observed in psychotherapy, where high psychological mindedness or curiosity may initially increase distress, but ultimately predict better long-term outcomes (Strupp, 1980). Likewise, exposure therapy increases fear and distress momentarily, but attenuates fear over time (Baker et al., 2010; Craske et al., 2014).

The current studies serve as replications and extensions of the research conducted by Nook et al. (2021), employing a similar methodology to further investigate the relationship between affect labeling and reappraisal. Building upon their findings, we introduce a novel hypothesis by incorporating an additional longer-term measurement. Specifically, we hypothesize that in the short-term (T1) affect labeling will cause a smaller decrease in unpleasant affect than reappraisal, whereas in the long-term (T2) affect labeling will be more effective in decreasing unpleasant affect than reappraisal. Additionally, we hypothesize that reappraisal will decrease unpleasant affect on both times of measurement.

## Method

### Study 1

This study’s desired sample size, variables, hypotheses, and planned analyses were preregistered on Open Science Framework (https://osf.io/j9624?view_only=a03e6afd6feb4649ac9b9fe5f4f52889) prior to any data being collected. The study was ethically approved by the Departmental Review Board.

#### Participants

For Study 1 we recruited 190 adults who participated in a study conducted through a questionnaire in Qualtrics for employees from the Amazon Mechanical Turk (MTurk) platform. We collected more participants than initially pre-registered (we pre-registered a stopping rule at 160) due to higher-than-expected drop-out rates. All participants were 18 or older, spoke fluent English, and obtained the “Mechanical Turk Masters” qualification for workers who have demonstrated excellent performance. Participants who completed the study received monetary compensation for their participation. Fifty-seven participants were eliminated for not completing session 1, not completing session 2, or completing either session more than once (further elaborated in the supplementary materials). The data of all eligible participants was coded by three independent judges, which led to further elimination of participants whose reappraisal responses were not valid, or whose affect labeling was not valid in more than a third of the trials (all elimination criteria pre-registered; an analysis including the eliminated participants according to external judges is provided in the supplementary materials). Note that we also removed specific trials on which the affect labeling was rated as invalid. Finally, we eliminated participants who rated all stimuli the same throughout a whole phase of the study. Thus, *a total number of 111 participants (pre-registered N = 117 wasn’t reach due to more ineligible participants than expected)* with valid responses were included in the final analysis (46% female, 54% male, age range = 21–76, *M*_age_ = 46.4, *SD*_age_ = 11.7). Following all exclusions, we remained with data of 30 participants in the Look condition, 24 in the Name condition, 28 in the Reappraise condition, and 29 in the Name & Reappraise condition for the final analysis.

#### Power Analysis

Following the replicated study by Nook et al. (2021), which detected a partial eta of .46 and used 80 participants, we used the software program G*Power 3.1 (Faul et al., 2009) to conduct a power analysis. Our goal was to obtain .95 power to detect a large effect size of .46 at the standard 0.05 alpha error probability for the first part of the study (T1). Given these parameters, the total number of participants required for the first part according to G*power was *n* = 88.

For the second part of the study (T2), we conducted a mixed-design analysis of variance (ANOVA). Our goal was to achieve a power of .95 to detect a medium effect size of .25, with an alpha level of 0.05, assuming sphericity correction would not be required. Power analysis suggested that a minimum of 60 participants was needed to carry out all three measurements. Given that the initial analysis required at least 81 participants and anticipating a high withdrawal rate due to multiple measurements, we aimed to recruit significantly more participants than the minimum necessary.

#### Experimental Paradigm and Procedure

**Fig. 1:**
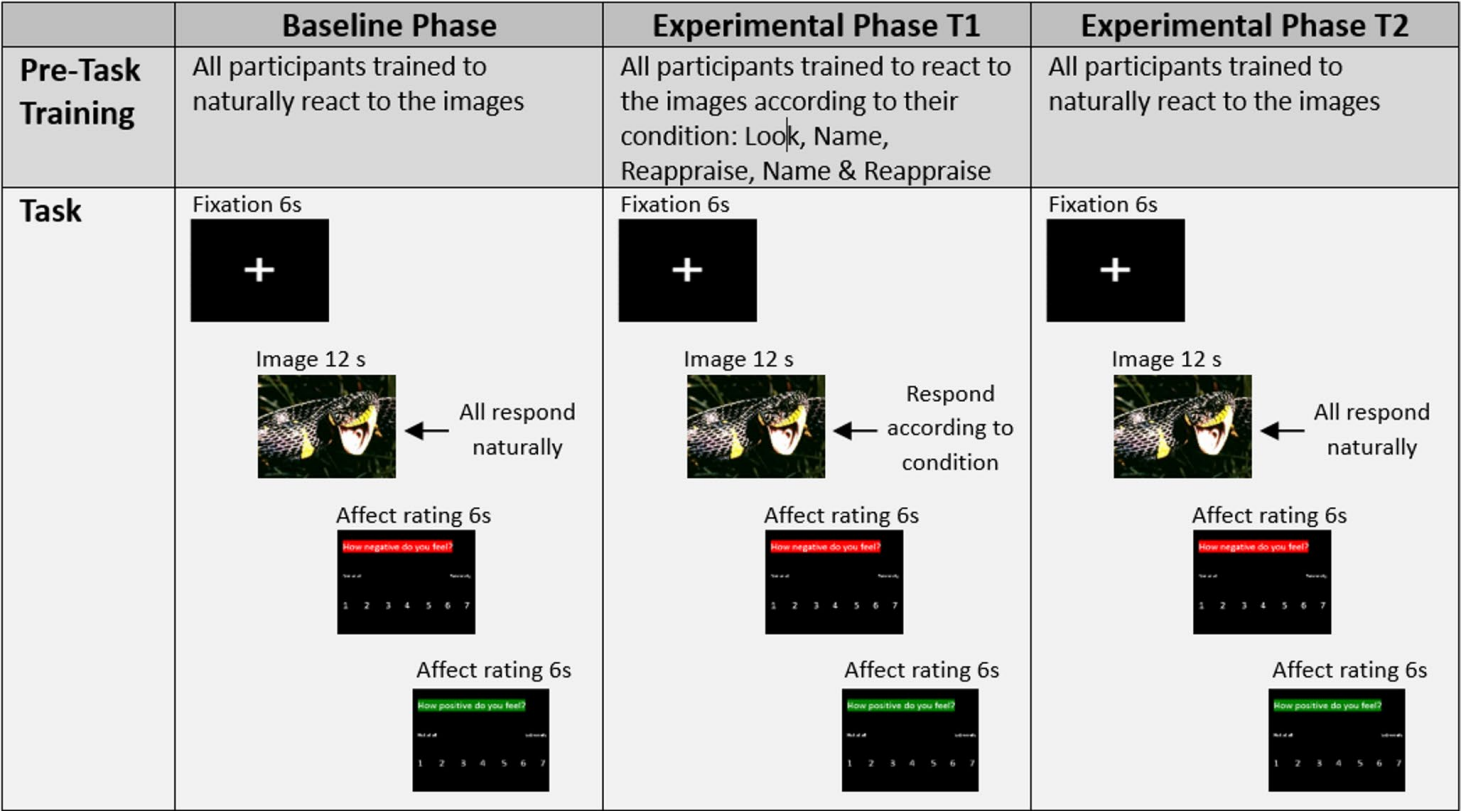
Description of all 3 phases of the experiment: Baseline phase, experimental phase T1, Experimental phase T2. Participants underwent training before each phase. In the baseline phase, participants viewed 24 negative images and responded to them naturally, then they were asked to rate the level of positive and negative emotion evoked by the images. In Experiment phase T1, participants were divided into four conditions, viewed the same 24 negative images again, and acted according to the different instructions given to each condition: (1) Passively observe the images (*Look*), (2) Label their most dominant emotion in light of the image (*Name*), (3) Regulate how they feel by creating a story about the image that makes them feel better (*Reappraise*), (4) Both label their dominant emotion and reappraise (*Name & Reappraise*). Afterwards, all participants rated their negative and positive emotions evoked by the images again. Experimental phase T2 was the same as the Baseline phase, all participants rated the same 24 images for the third time with no extra instructions provided

We conducted a replication of the experimental paradigm used by Nook et al. (2021) in their Study 1 to test how affect labeling impacts emotion reappraisal, and added another phase to it that allowed us to test our hypothesis regarding the lasting effects of affect labeling (note that in their study, Nook et al. (2021) refer to affect labeling as ‘emotion naming’) (Fig. 1). The paradigm was based on reinterpretation, one of the two variations of reappraisal that were employed in the original paradigm as an emotion regulation strategy (McRae et al., 2012). Following informed consent, all participants completed three phases.

The baseline phase contained 24 trials, in which participants were asked to passively watch a negatively valenced image from the International Affective Image Set (IAPS; Lang et al., 2005). Following each image, participants had to rate how negative and positive they felt in response to the image using separate 1–7 scales (each asking about negative or positive affect), with 1 being ‘not at all’ and 7 being ‘a great deal’ (Kron et al., 2013). The order of the negative and positive scales was counterbalanced across trials, and the order of images was randomized across participants and phases. Valence norms for the images ranged from moderately to extremely negative (range = 1.6–3.95 on a 1–9 scale where one is most negative, *M*_valence_ = 2.68, *SD*_valence_ = .63), and arousal norms ranged from moderately to highly arousing (range = 4.14–7.09 on a 1–9 scale where nine is most arousing, *M*_arousal_ = 5.69, *SD*_arousal_ = .78; the IDs of the presented pictures are provided in the supplementary materials).

The purpose of the baseline phase was to set baseline responses to the images so that variations in affective responses to the images under each manipulation carried out in the subsequent phase could be observed and compared.

Subsequently, participants completed the first experimental phase (T1), in which they were randomly assigned to one of four between-participants conditions, using a 2 [Affect Labeling: Not Labeling vs. Labeling] x 2 [Reappraising: Not Reappraising vs. Reappraising] study design. Similar to the baseline phase, there were 24 trials, and participants rated their positive and negative affect after each image (see supplementary materials for full task instructions per condition).

In the first condition, *Look* (not affect labeling + not reappraising), participants were asked to passively observe the images and rate them for the second time, similar to the baseline phase. This condition was meant to evaluate the effect of re-exposure alone on affect ratings. In the second condition, *Name* (affect labeling + not reappraising), participants were asked to write the emotion they most strongly felt as each image appeared on the screen. Here we were forced to deviate from the original paradigm due to technical constraints, since the study was conducted online, and we could not ensure participants had the means to audio record themselves. Instead, participants typed in their emotions. However, studies show that both writing and verbalizing used as expressive disclosure have therapeutic benefits on distress and well-being (Frattaroli, 2006; Pennebaker, 1997; Pennebaker & Beall, 1986). Therefore, we did not expect this alteration to affect the results. We compared the *Look* and *Name* conditions to assess the impact of affect labeling on its own.

In the third condition, *Reappraise* (not affect labeling + reappraising), participants were asked to regulate their emotional reactions to the images using the reappraisal regulation strategy (note that in their study, Nook et al. (2021) refer to reappraise as ‘regulate’). They did so through reinterpretation, i.e., a reappraisal technique that involves providing a new context to the image that makes it less arousing. Lastly, the fourth condition, *Name & Reappraise* (affect labeling + reappraising), included the same instructions from both the *Name* and *Reappraise* conditions, in that order. These two last conditions allowed us to compare them and test how affect labeling impacts reappraisal. In all phases, each trial lasted 30 s, structured as follows: 6 s for a fixation cross, 12 s for image presentation and response (which varied by condition), 6 s for the positive affect rating, and 6 s for the negative affect rating (scale order counterbalanced across trials). Each phase required approximately 12 min to complete.

Finally, we added a second experimental phase (T2) with 24 trials, creating a new within-participants condition: time of measurement [T1] x [T2]. This phase (T2) was administered between one to two days after completing the previous assignments (i.e., baseline phase and first experimental phase) and entailed the same instructions as the baseline phase did, as well as the same images, presented in randomized order. The purpose of this phase was to put our hypotheses to the test by comparing participants’ ratings from the baseline phase to their ratings on T2.

To verify comprehension of tasks, participants completed a series of practice trials before each of the three phases. We adopted an inclusive strategy, similar to that of Nook et al. (2021), allowing participants to create their own emotional terms rather than selecting from a limited list of emotions (for the full list of emotions used by participants see Supplementary Materials). Answers that addressed the image’s content rather than the emotion it evoked, however, were non-compliant and excluded from the analysis (as rated by 3 independent judges, an emotion was deemed invalid if at least 2 of the three judges rated it as such; interrater reliability: *ICC*_(2,3)_ = .909, *F*_(1655,3310)_ = 11.34, *p* < 0.001, 95% *CI* [.89, .91]).

Furthermore, to ensure that participants in the Reappraise and Name & Reappraise conditions genuinely engaged in cognitive reappraisal, we included three additional trials at the end of the study. In these trials, participants were asked to write the story they used to reappraise their emotional response to three previously seen images. These responses were independently evaluated by three independent judges. A response was deemed valid if it (1) reflected the generation of a novel interpretation of the image and (2) conveyed a change in meaning that could potentially improve the participant’s emotional experience. Only responses validated by at least two of the three raters were included (*ICC*_(2,3)_ = .85, *F*_(233,466)_ = 7.57, *p* < 0.001, 95% *CI* [.81, .89]).

#### Analysis

For the replication analysis, we employed the same ANOVA and *t*-test analysis as in the original study by Nook et al. (2021). Prior to the analysis we computed our dependent variable “change in unpleasant affect” (*∆ unpleasant affect T1*). For each trial we calculated the unpleasant affect as the average of the negative scale and the reversed positive scale, then we subtracted the participant’s mean baseline-phase unpleasant affect (over all images) from their mean experimental-phase T1 unpleasant affect.

For the first part of the study, we employed a 2 [Affect Labeling: Not Labeling vs. Labeling] x 2 [Reappraising: No Reappraising vs. Reappraising] ANOVA with two between-subjects factors. Then, we conducted two independent samples *t*-tests with the dependent variable ∆ unpleasant affect to compare between: (1) *Reappraise* and *Name & Reappraise* conditions and (2) between *Name* and *Look* conditions. Finally, we conducted 4 single-sample *t*-tests, comparing the dependent variable ∆ unpleasant affect to zero in each combination of the two factors (the 4 conditions: *Look*, *Name*, *Reappraise*, *Name & Reappraise*).

For the second part of the study (T2), we used a mixed linear model with fixed effects for Reappraise (effect coded; no reappraisal = -1, reappraisal = 1), Name (effect coded; no naming = -1, naming = 1), Time of measurement (effect coded; Baseline = -1, T1 = 0, T2 = 1), and their interactions. The model included random intercepts and random slopes of Time of Measurement for participants and for images.

### Study 2

Study 2 was an exact replication of Study 1 with two minor changes to ensure the robustness Study 1’s findings. Study 2 was pre-registered prior to data collection (https://osf.io/2vq4z? view_only=4b74ad04be8e4904b413a6ddc81f96b1) with revised hypotheses derived from the results of Study 1. Study 2 was ethically approved by the Departmental Review Board. The data and analysis codes for both studies can be found in the OSF project (https://osf.io/bvkxf/?view_only=4b74ad04be8e4904b413a6ddc81f96b1).

#### Participants

For Study 2 we aimed to recruit a similar sample size as in Study 1. For that end, we recruited 162 Participants from Prolific. Participants were 18 years old or more, fluent in English, and had a Prolific quality rating of 95% or higher with a history of completing at least 20 studies on Prolific. Participant exclusion followed the same principles as in Study 1 (all exclusion criteria were pre-registered), removing all participants who did not finish either one of the sessions, who did not return in time for session 2, or who have begun one of the sessions more than once. Of the eligible participants, we further excluded participants who have failed the reappraisal quality check (interrater reliability: *ICC*_(2,3)_ = .84, *F*_(227,454)_ = 7.24, *p* < 0.001, 95% *CI* [.80, .88]), participants who have provided invalid emotion naming in more than a third of their trials (interrater reliability: *ICC*_(2,3)_ = .89, *F*_(1823,3464)_ = 10.02, *p* < 0.001, 95% *CI* [.88, .90]), and (as an addition to Study 1) participants who understood the purpose of the study as judged by 3 independent judges (interrater reliability: *ICC*_(2,3)_ = .66, *F*_(152,152)_ = 3.08, *p* < 0.001, 95% *CI* [.52, .75]). Finaly we excluded participants who rated all 24 images in a phase (including attention check images) with the same values. All exclusions are elaborated in the supplementary materials. The judges who scored the reappraisal quality, the emotion validity and the study’s purpose were the same judges in both studies. An analysis including the participants excluded due to the judges’ rating is presented in the supplementary materials. Note that we also removed specific trials on which the affect labeling was rated as invalid.

The final analyzable sample included *N* = 115 participants (45% female, 54% male, 1 participant identified as Genderqueer / Gender non-conforming; age range = 21–76, *M*_age_ = 43.2, *SD*_age_ = 13.9), 35 of which were randomly allocated to the *Look* condition, 35 to the *Name* Condition, 21 to the *Reappraisal* condition, and 24 to the *Name & Reappraisal* condition.

#### Experimental Paradigm, Procedure and Analysis

The experiment was an exact replication of Study 1 with minor modifications. First, to differentiate between participants providing genuine emotional ratings to inattentive participants providing uniform response to all images, we added four images to each phase (Baseline, Experimental T1, and Experimental T2) as attention checks. Specifically, in the Baseline phase, four neutral pictures were added in random locations, and on the Experimental phases T1 and T2, two of the neutral were added at random location and extremely positive pictures were anchored at the end of the session to allow participants to leave the study on a positive note.

The second change involved the addition of an open-ended question at the end of the study, probing for the participants’ thoughts on the study and of its purpose. The question also included a hidden prompt with altered instructions (invisible to human readers) to detect automated responses (e.g., by bots); no suspicious entries were found.

## Results

**Fig. 2:**
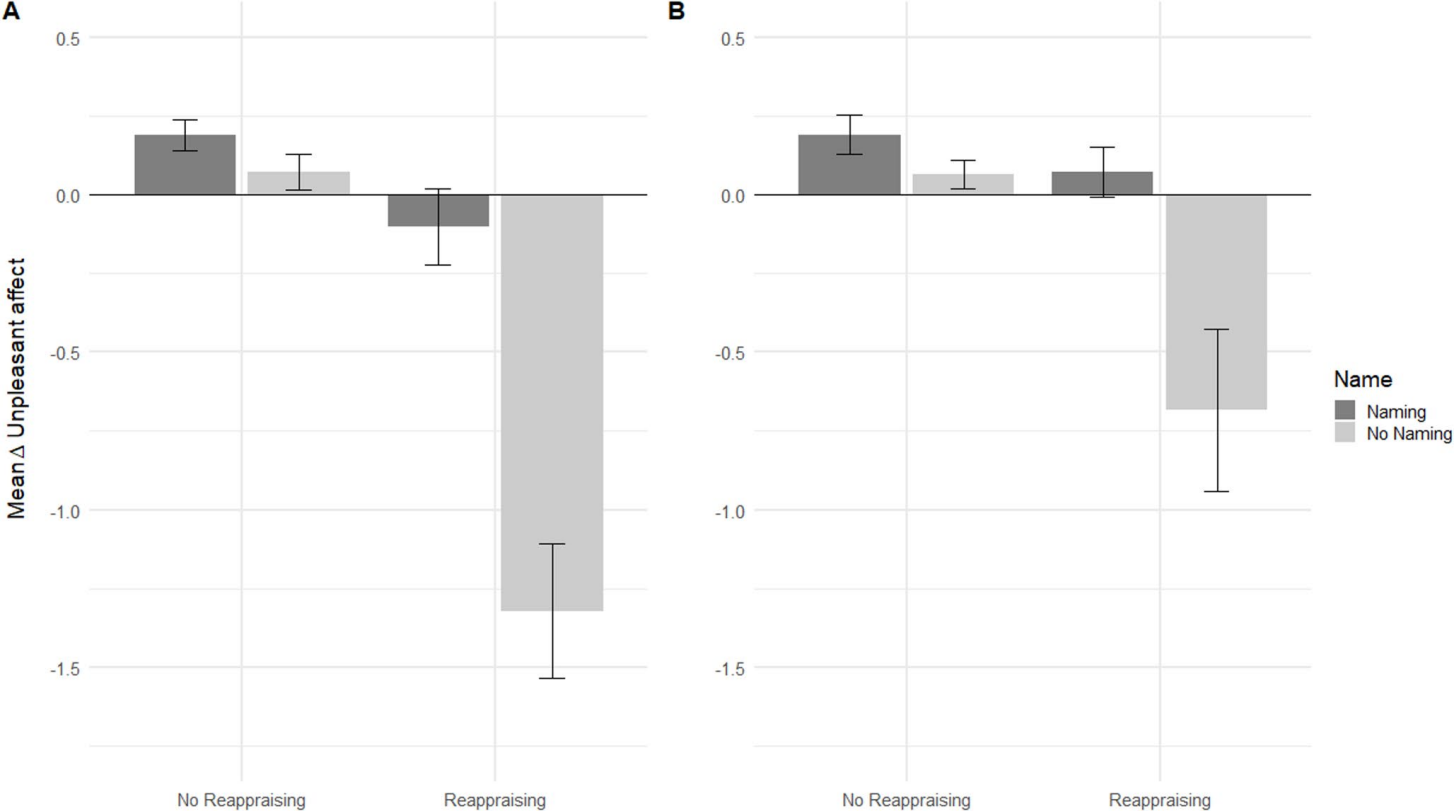
Displays the mean change in unpleasant affect (∆ unpleasant affect) from baseline to T1 for each of the four experimental conditions for Study 1 (panel A) and Study 2 (panel B). The comparison between the *Look* and *Name* conditions did not yield a significant difference, indicating that naming alone does not impact the emotional response to the image significantly. However, a significant difference was observed between the *Reappraise* and *Name & Reappraise* conditions, suggesting that naming before engaging in reappraisal impedes the regulation of emotional response to the image, in contrast to reappraisal alone

In Study 1, the replication analysis supported the results from the original study by Nook et al. (2021). We found that reappraisal had a main effect on ∆ unpleasant affect, suggesting that reappraisal helped participants decrease their unpleasant affect (*M* = -.7, *SD* = 1.10), in comparison to not reappraising (*M* = .12, *SD* = .28); *F*_(1,107)_ = 44.29, *p* < 0.001, *η*_*p*_^*2*^ = .29, 95% *CI* [.18, 1.00]). Moreover, affect labeling was found to have a main effect on ∆ unpleasant affect; that is, participants who did not label experienced less negative affect (*M* = -.6, *SD* = 1.07) than those who did label (*M* = 0.03, *SD* = .52; *F*_(1,107)_ = 23.31, *p* < 0.001, *η*_*p*_^*2*^ = .18, 95% *CI* [0.08, 1.00]). A significant interaction was observed between affect labeling and reappraising (*F*_(1,107)_ = 17.08, *p* < 0.001, *η*_*p*_^*2*^ = .14, 95% *CI* [0.06, 1.00]; see Fig. 2.A).

These results were replicated in the ANOVA on the data from Study 2, such that reappraisal reduced unpleasant affect (*M* = -.28, *SD* = .92) compared to not reappraising (*M* = .12, *SD* = .33; *F*_(1,111)_ = 13.91, *p* < 0.001, *η*_*p*_^*2*^ = .11, 95% *CI* [0.04, 1.00]). Affect labeling increased unpleasant affect (*M* = .14, *SD* = .38) compared to no affect labeling (*M* = -.21, *SD* = .82; *F*_(1,111)_ = 10.67, *p* = 0.001, *η*_*p*_^*2*^ = 0.09, 95% *CI* [0.02, 1.00]). And their interaction was also significant (*F*_(1,111)_ = 7.81, *p* = 0.006, *η*_*p*_^*2*^ = 0.07, 95% *CI* [0.01, 1.00]; see Fig. 2.B).

Independent samples *t*-tests (Welch’s *t*-test utilized due to unequal variances) on data from both studies replicated the finding of Nook et al. (2021). Reappraisal alone (Study 1: *M* = -1.32, *SD* = 1.13; Study 2: *M* = -.68, *SD* = 1.18) significantly reduced ∆ unpleasant affect compared to engaging in reappraisal following affect labeling (Study 1: *M* = -.1, *SD* = .65, *t*_(42.94)_ = 4.97, *p* < 0.001, *d* = 1.32, 95% *CI* [.73, 1.91]; Study 2: *M* = 0.07, *SD* = .38, *t*_(23.7)_ = 2.8, *p* = 0.009, *d* = .86, 95% *CI* [.21, 1.5]). On the other hand, engaging in affect labeling did not significantly change the unpleasant affect (Study 1: *M* = .19, *SD* = .24; Study 2: *M* = .19, *SD* = .37) compared to simply observing the image without engaging in any regulation strategy (Study 1: *M* = 0.07, *SD* = .3, *t*_(51.99)_ = 1.53, *p* = .129, *d* = .42, 95% *CI*[-.12, .95]; Study 2: *M* = 0.06, *SD* = .27, *t*_(61.91)_ = 1.62, *p* = .109, *d* = .39, 95% *CI* [-0.09, .86]).

Finally, the outcomes of one-way *t*-tests diverged somewhat from those reported by Nook et al. (2021). In both the *Look* and *Name & Reappraise* conditions, the level of ∆ unpleasant affect did not exhibit significant deviation from 0, indicating that participants did not manifest alterations in their affective responses under these conditions (*Look*: Study 1: *t*_(29)_ = 1.3, *p* = .203, *d* = .24, 95% *CI* [-.13, .6]; Study 2: *t*_(34)_ = 1.39, *p* = .17, *d* = .24, 95% *CI* [-.1, .57]; *Name & Reappraise*: Study 1: *t*_(28)_ = -.84, *p* = .407, *d* = -.16, 95% *CI* [-.52, .21]; Study 2: *t*_(23)_ = .92, *p* = 0.366, *d* = .19, 95% *CI* [-.22, .59]). Conversely, in the *Name* condition, there was an increase in ∆ unpleasant affect, suggesting that not only did affect labeling fail to regulate participants’ affect, but it exacerbated it (Study 1: *t*_(23)_ = 3.75, *p* = 0.001, *d* = .77, 95% *CI* [.3, 1.22]; Study 2: *t*_(34)_ = 3.01, *p* = 0.004, *d* = .51, 95% *CI* [.15, .86]). Ultimately, it was solely in the *Reappraise* condition that a decrease in ∆ unpleasant affect from 0 was observed, underscoring the efficacy of reappraising as the sole effective strategy for modulating participants’ affective responses (Study 1: *t*_(27)_ = -6.19, *p* < 0.001, *d* = -1.17, 95% *CI* [-1.65, -.68]; Study 2: *t*_(20)_ = -2.65, *p* = 0.015, *d* = -.58, 95% *CI* [-1.04, -.11]).

**Fig. 3:**
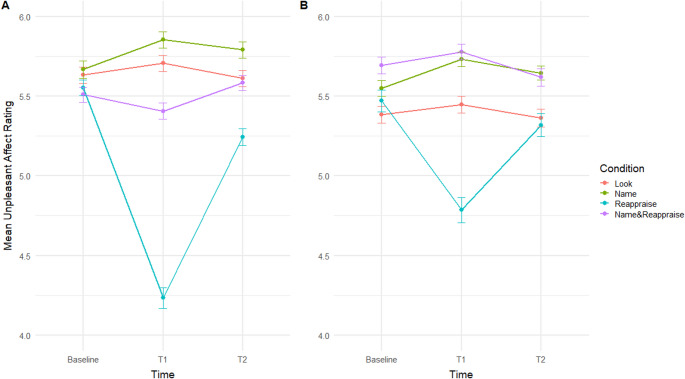
The means of unpleasant affect under each condition and time of measurement (Baseline, T1, T2) for (**A**) Study 1 and (**B**) Study 2. Error bars indicate standard errors

Subsequently, to evaluate our primary hypothesis, we conducted an analysis of unpleasant affect using a mixed linear model with 3 times of measurement on the data of both studies. The analysis revealed a significant three-way interaction between reappraisal, naming, and time of measurement (for the results of the full model see Table 1). Deconstructing this interaction by examining the two main effects (reappraise and name) and their interaction at each timepoint, we found that at T1 there were significant effects for Naming, Reappraising (significant in Study 1) and their interaction (as expected from the ANOVA analysis reported above). However, and in contrast to our pre-registered hypotheses for Study 1 but fully supporting the pre-registered hypotheses for Study 2, neither Naming, Reappraisal or their interaction exerted any significant effects at Baseline and T2 (see Table 2; Fig. 3). Notably, the lack of significant effects at T2 in both studies suggests that the significant effects of reappraisal on reducing unpleasant affect observed in T1 are short lived.

**Table 1:** The results of the mixed linear model employed in both studies

	Study 1					Study 2				
	df1	df2	F	*p*	η_*p*_^2^	df1	df2	F	*p*	η_*p*_^2^
Reappraise	1	107.06	11.3	0.001	.10	1	111	.26	.61	< 0.01
Name	1	107.06	4.64	0.033	0.04	1	111	5.82	0.017	0.05
Time	2	100.72	10.08	< 0.001	.17	2	102.14	1.15	.318	0.02
Reappraise * Name	1	107.06	2.07	.150	0.02	1	111	.71	.4	< 0.01
Reappraise * Time	2	108.28	20.54	< 0.001	.28	2	111.12	7.3	0.001	.12
Name * Time	2	108.28	14.26	< 0.001	.21	2	111.12	7.88	< 0.001	.12
Reappraise * Name *Time	2	108.28	8.86	< 0.001	.14	2	111.12	5.36	0.005	0.09

Results are from Type III tests of fixed effects with Satterthwaite’s approximation for denominator degrees of freedom (lmerTest package)

**Table 2:** Main effects and interactions within each time of measurement for both studies

Time of Measurement	Effect	Study 1				Study 2			
		**df1**	**df2**	**F**	***p***	**df1**	**df2**	**F**	***P***
Baseline	Reappraise	1	∞	.77	.379	1	∞	.53	.465
	Name	1	∞	0.03	.849	1	∞	1.58	.208
	Reappraise * Name	1	∞	0.06	.804	1	∞	0.04	.83
T1	Reappraise	1	∞	32.45	< 0.001	1	∞	3.35	0.066
	Name	1	∞	14.84	< 0.001	1	∞	13.92	< 0.001
	Reappraise * Name	1	∞	9.75	0.001	1	∞	4.19	0.04
T2	Reappraise	1	∞	3.48	0.061	1	∞	0.05	.815
	Name	1	∞	2.59	.107	1	∞	2.93	0.086
	Reappraise * Name	1	∞	.32	.556	1	∞	0.00	.952

Tests with denominator df = ∞ are Wald-type χ² tests provided by emmeans::joint_tests

## Discussion

Our investigation aimed to investigate the role of affect labeling in influencing emotion regulation outcomes, with a particular focus on the timing of measurement. Our findings across both studies bolster the conclusions drawn by Nook et al. (2021) regarding the initial assessment of affect labeling, illustrating that affect labeling hinders reappraisal as an emotion regulation strategy. Specifically, participants who engaged in affect labeling prior to regulation reported experiencing more negative affect compared to those who regulated without preceding affect labeling. Contrary to our initial hypothesis, the timing of measurement did not emerge as a significant factor influencing the impact of affect labeling on regulation outcomes. Notably, participants’ responses to the images on T2 resembled their reactions during the initial measurement, i.e., to baseline levels.

Interestingly, we also found that reappraisal, which was effective in reducing negative affect at T1—did not differ from baseline at T2, suggesting that its effects were not sustained over time. This finding adds to a growing body of research questioning the durability of emotion regulation strategies over time. For instance, Denny and Ochsner (2014) demonstrated that longitudinal training in reappraisal can lead to better ability to regulate, whereas no training doesn’t lead to comparable gains. In a similar vein, Sahi et al. (2023) found that social reappraisal, i.e., reappraisal guided by peers, can yield longer-lasting effects: participants who engaged in social reappraisal exhibited reductions in negative affect that persisted even 24 h after regulation. In contrast, participants who performed reappraisal on their own showed a return to baseline affective responses at follow-up, a pattern that aligns with our own findings. Their findings, together with our results, highlight the importance of examining both immediate and longer-term consequences of emotion regulation strategies. However, it is important to note a limitation in our study design: the instructions given at T2 were identical to those provided during the baseline phase. It is possible that the return to baseline levels of negative affect at T2 may be due, at least in part, to this repetition of instructions rather than a true decline in the regulatory effects. Future research could address this issue by employing differently worded instructions at the two phases to better isolate regulatory effects over time.

Our additional findings regarding the effects of affect labeling on emotion regulation further underscore the complexity of this process. Despite the efforts to elucidate the underlying mechanisms, the precise way in which affect labeling influences emotional responses remains elusive, and discrepancies within this realm of investigation persist. Explanations for these discrepancies were given by Nook et al. (2021) and were mentioned here before. Yet, it is also plausible that methodological limitations inherent in our study design may have hindered the detection of temporal fluctuations in the effects of affect labeling on regulation outcomes. Perhaps a different study design could yield different results, for example a study wherein the timing of regulation relative to affect labeling will be altered, such that regulation will occur only after a one-day interval following affect labeling rather than immediately following labeling as in our current protocol. This approach would allow to test again our hypothesis regarding the timing of measurement, potentially revealing that the regulation strategy should be executed only after the impact of labeling has initiated (Thiruchselvam et al., 2011).

Additionally, technical constraints precluded participants from verbally articulating their emotions aloud, which may have influenced the outcomes obtained. It is possible that this mode of responding—typing the emotion word rather than speaking it- contributed to the unexpected finding that affect labeling alone increased negative affect. According to a recent meta-analysis (Guo, 2023), expressing emotions through writing yields positive long term psychological effects in reducing symptoms of depression, anxiety and stress. Yet, short-term effects are generally insignificant and can even lead to negative outcomes such as increase in distress, negative mood and physical symptoms, and a decrease in positive mood (Baikie & Wilhelm, 2005; Torre & Lieberman, 2018). Seeing one’s emotional state explicitly written out may reinforce or consolidate the emotional experience, rather than diffusing it. Prior work has shown that writing about emotions can intensify the accessibility and structure of emotional memories (Pennebaker, 1997), potentially amplifying the salience of the emotional response in the short term. These negative short-term psychological effects of written affect labeling line up with our findings and might explain the increase in negative mood immediately after writing ones emotion elicited by the pictures.

Furthermore, our findings underscore the need for future research to explore alternative explanations for the mechanism of affect labeling and its influence on emotion regulation. While our study focused primarily on the temporal dynamics of affect labeling, it is possible that other factors, such as individual differences in emotional processing or contextual factors, may moderate the effects of affect labeling on regulation outcomes (Vine et al., 2019). Investigating these potential moderators may provide valuable insights into the conditions under which affect labeling is most effective in promoting emotion regulation.

An alternative perspective suggests that future research endeavors should strive for greater ecological validity to more accurately capture the role of affect labeling. For instance, stimuli utilized in studies should be personally relevant and emotionally salient to each participant, rather than standardized across participants. Furthermore, recent studies have shown that social forms of reappraisal—where individuals receive interpretive support from peers—lead to stronger and more enduring reductions in negative affect compared to solitary regulation (Sahi et al., 2023). These findings highlight the natural tendency of individuals to regulate emotions through social interaction and suggest that affect labeling, when embedded in relational contexts, may more closely resemble its everyday use and thereby enhance its regulatory utility.

Beyond its applied implications, the present findings contribute to a broader understanding of affect labeling as a fundamental emotion process. Within affective science, affect labeling has been conceptualized as a mechanism through which language interacts with core affect to shape emotional experience (Barrett, 2006; Lindquist et al., 2015). Our results reinforce this view by demonstrating that labeling one’s affective state can influence subsequent emotional dynamics—even outside explicit regulatory intent. As such, affect labeling warrants continued investigation as a naturally occurring component of emotion construction and modulation.

In conclusion, our study contributes to a growing body of literature examining the role of affect labeling in emotion regulation. By investigating the temporal dynamics of affect labeling and its impact on regulation outcomes, we have uncovered novel insights into the complexities of emotion regulation processes. However, further research is needed to fully elucidate the mechanisms underlying the influence of affect labeling on regulation outcomes and to identify the conditions under which affect labeling is most beneficial.

## Supplementary Information

Below is the link to the electronic supplementary material.

Supplementary Material 1 (DOCX 183 KB)

## Additional Information

### Data Availability

The data and analysis codes for both studies can befound in the OSF project (https://osf.io/bvkxf/viewonly=4b74ad04be8e4904b413a6ddc81f96b1).
