# Supplementary material for: Affect Labeling and Reappraisal as an Emotion Regulation Strategy
Source: Affect Sci. 2026 Mar 30;7(2):282–92. doi: 10.1007/s42761-026-00362-z (PMC13269579; doi:10.1007/s42761-026-00362-z)

**Supplementary Materials**

To accompany

Affect labeling and Cognitive Reappraisal as Emotion Regulation Strategy

Yael Ariely1ᶧ, Aviv Mokady1,^2^ᶧ, Niv Reggev^1,2^, Gideon Anholt1

Ben-Gurion University of the Negev, Faculty of Humanities and Social Science, Department of Psychology, Beer Sheva, Israel.

^2^ Ben-Gurion University of the Negev, School of Brain Sciences and Cognition, Beer Sheva, Israel.

ᶧ Denotes equal contribution; the order of authorship among equal contributors was determined randomly using a digital application.

ORCID identifiers:

Aviv Mokady: 0000-0003-4475-0332

Gideon E. Anholt: 0000-0002-5128-239

Niv Reggev: 0000-0002-5734-7457

Table of Contents

1. Task instructions
2. IAPS images IDs
3. Study 1
   1. Participant Exclusion Details
   2. Affect labels generated by participants
   3. Negative and Positive Affect Rating Distributions
   4. Analyses of Δ negative and Δ positive affect
   5. Results with excluded participants
4. Study 2
   1. Participant Exclusion Details
   2. Affect labels generated by participants
   3. Negative and Positive Affect Rating Distributions
   4. Analyses of Δ negative and Δ positive affect
   5. Results with excluded participants

**I. Task Instructions**

**Baseline Phase.** For this phase, you will see a series of images. Simply observe each image while it is on the screen. After each image, rate how you feel on the scales provided.

**Look Condition.** For this phase, you will see a series of images. Simply observe each image while it is on the screen. After each image, rate how you feel on the scales provided.

**Name Condition.** For this phase, you will see a series of images. While each image is on the screen, decide what is the most dominant emotion you feel, and type the name of this emotion in the space provided. Please use only a single word or very short phrase. If it is ever difficult to name what you are feeling, keep trying until you do so or until the image disappears. After each image, rate how you feel on the scales provided.

**Regulate Condition.** For this phase, you will see a series of images. While each image is on the screen, regulate how you feel by creating a story about the image that makes you feel better about it. After each image, rate how you feel on the scales provided.

**Name and Regulate Condition.**  For this phase, you will see a series of images. While each image is on the screen, first decide what is the most dominant emotion you feel, and type the name of this emotion in the space provided. Please use only a single word or very short phrase. If it is ever difficult to name what you are feeling, keep trying until you do so or until the image disappears. Second, regulate how you feel by creating a story about the image that makes you feel better about it. After each image, rate how you feel on the scales provided.

**II. IAPS images ID's**

**Practice trials:** 9912, 9810, 9921, 2100, 1932, 1270

**Experimental trials:** 1052, 1200, 1525, 2053, 2683, 2981, 3051, 3100, 3220, 3300, 6244, 6550, 6555, 6838, 7380, 9010, 9041, 9181, 9280, 9301, 9561, 9570, 9600, 9630, 9635.1

**Attention check trials:** 1333, 2515, 2358, 1942, 1710, 1750

**III. Study 1**

**III.a. Participant Exclusion**

One-hundred and ninety participants began the study on the first day. Fifty-seven participants were excluded from data analysis for the following reasons:

- Eighteen participants took the task on the first day twice (some did not complete both attempts, but because stimuli were seen more than intended, these participants were excluded).
- Twenty-one participants did not complete the task on the first day.
- Nine did not return or did not finish the task on the second day.
- Six participants completed the task on the second day twice (some did not complete both attempts, but because stimuli were seen more than intended, these participants were excluded).
- Three participants did not complete the task on the second day.

We ended up with N = 133 participants with eligible entries. The data of these participants was coded for valid reappraisal and valid affect labeling by 3 independent judges, which led to the exclusion of:

- Sixteen participants failed to provide valid reappraisals.
  - Six from the Name & Reappraise condition and 10 from the Reappraise condition
- Three participants failed to provide valid affect labeling in more than a third of the trials.
- Three participants rated all 24 images exactly the same on both the negative and the positive scales throughout a whole session.

The final sample reported in the manuscript after all exclusions included N = 111 participants.

**III.b. Affect labels Generated by Participants**

The table displays the original entries by participants; only undoubted spelling errors were corrected.

| **Quantity** | **Affect label** |
| --- | --- |
| 1 | Frustration |
| 1 | Abhorred |
| 13 | Afraid |
| 3 | Aggression |
| 1 | Aggression Fear |
| 1 | Aggressive |
| 2 | Agitated |
| 2 | Agony |
| 5 | Alarm |
| 2 | Alarmed |
| 1 | Amazed |
| 5 | Amazement |
| 1 | Ambivalent |
| 40 | Anger |
| 1 | Anger Agression |
| 20 | Angry |
| 1 | Angry And Fear And Good |
| 1 | Angst |
| 2 | Anguish |
| 1 | Anguished |
| 4 | Annoyed |
| 1 | Anticipation |
| 6 | Anxiety |
| 5 | Anxious |
| 3 | Anxiousness |
| 1 | Apalling |
| 1 | Apathetic |
| 5 | Apathy |
| 3 | Appalled |
| 3 | Apprehension |
| 1 | Arrogant |
| 1 | Astonish |
| 1 | Aversion |
| 1 | Avoidant |
| 4 | Awe |
| 1 | Awe Angst |
| 2 | Awestruck |
| 1 | Awful |
| 1 | Bad |
|  |  |
|  |  |
| **Quantity** | **Affect label** |
| 1 | Bad Luck |
| 1 | Benign |
| 2 | Bitter |
| 1 | Boredom |
| 1 | Brave |
| 1 | Bravery |
| 2 | Broken Hearted |
| 7 | Calm |
| 1 | Captive |
| 1 | Care |
| 1 | Caution |
| 1 | Cautious |
| 13 | Compassion |
| 1 | Compassion Want To Help |
| 7 | Concern |
| 1 | Concern Helplessness |
| 8 | Concerned |
| 2 | Confined |
| 5 | Confused |
| 2 | Confusion |
| 1 | Creeped Out |
| 3 | Creepy |
| 1 | Cruel |
| 1 | Cruelty |
| 1 | Crushed |
| 1 | Crying |
| 7 | Curiosity |
| 10 | Curious |
| 1 | Curioy |
| 7 | Depressed |
| 2 | Despair |
| 1 | Despise |
| 1 | Detestable |
| 1 | Detestion |
| 3 | Devastated |
| 2 | Digust |
| 2 | Dirty |
| 2 | Disappointed |
| 1 | Disappointm |
|  |  |
|  |  |
| **Quantity** | **Affect label** |
| 122 | Disgust |
| 1 | Disgust At Our Pollution |
| 24 | Disgusted |
| 19 | Disgusting |
| 1 | Disgusting Unhygin |
| 1 | Disinterest |
| 1 | Dismay |
| 1 | Dread |
| 1 | Dreadful |
| 1 | Drowning |
| 4 | Empathetic |
| 12 | Empathy |
| 1 | Excited |
| 2 | Exiting |
| 156 | Fear |
| 1 | Fear Agression |
| 1 | Fear And Sadness |
| 1 | Fear Apprehension |
| 1 | Fear Factor |
| 10 | Fearful |
| 1 | Fearful Sad |
| 1 | Fearless |
| 1 | Fearsome |
| 1 | Feeling |
| 1 | Fierce Danger |
| 1 | Fine |
| 1 | Forceful |
| 1 | Freakd Out |
| 1 | Fright |
| 6 | Frightened |
| 1 | Funny |
| 1 | Furious |
| 1 | Gloomy |
| 2 | Grief |
| 2 | Grimm |
| 1 | Grimm Worriy |
| 17 | Gross |
| 9 | Grossed Out |
| 1 | Happiness |
| 2 | Happy |
| 1 | Harmful |
| 3 | Hate |
|  |  |
|  |  |
| **Quantity** | **Affect label** |
| 2 | Hateful |
| 1 | Heart Breaking |
| 4 | Heartbroken |
| 6 | Helpless |
| 1 | Hesitant |
| 4 | Hope |
| 1 | Hopeful |
| 4 | Hopeless |
| 1 | Hopelessness |
| 3 | Horrible |
| 2 | Horrific |
| 15 | Horrified |
| 7 | Horror |
| 2 | Hurt |
| 1 | Hurting |
| 2 | Indifference |
| 2 | Indifferent |
| 1 | Indignant |
| 1 | Infuriatin |
| 1 | Inhuman Sorrow |
| 1 | Intens |
| 9 | Interest |
| 3 | Interested |
| 1 | Interesting |
| 1 | Intrigue |
| 1 | Intrigued |
| 1 | Introspective |
| 1 | Irritable |
| 1 | Irritated |
| 7 | Irritating |
| 6 | Irritation |
| 1 | Lonely |
| 1 | Lost |
| 1 | Love |
| 1 | Lovely |
| 5 | Mad |
| 1 | Maddened |
| 1 | Mild Anxiety |
| 1 | Mild Disgust |
| 2 | Mild Sadness |
| 3 | Miserable |
| 1 | Mournful |
| 2 | Nasty |
|  |  |
| **Quantity** | **Affect label** |
| 1 | Natural |
| 1 | Nature Disaster |
| 3 | Nausea |
| 4 | Nauseous |
| 2 | Negative |
| 12 | Nervous |
| 12 | Neutral |
| 1 | No Hope |
| 3 | Normal |
| 1 | Offence |
| 1 | Ok |
| 1 | On Edge |
| 2 | Outrage |
| 3 | Pain |
| 1 | Pained |
| 8 | Painful |
| 4 | Panic |
| 1 | Panicked |
| 8 | Pathetic |
| 1 | Pissed |
| 1 | Pissed Off |
| 19 | Pity |
| 1 | Polluted |
| 1 | Preoccupied |
| 2 | Pride |
| 1 | Psychotic |
| 3 | Puzzlement |
| 2 | Queasy |
| 3 | Rage |
| 1 | Relief |
| 3 | Repulsed |
| 1 | Restricted |
| 2 | Revolted |
| 1 | Revolting |
| 4 | Revulsion |
| 140 | Sad |
| 1 | Sad Grimm |
| 1 | Sad Moments |
| 1 | Sad Sorrow |
| 1 | Sad Sorrow Pity |
| 1 | Sad Very |
| 1 | Sad compassionate |
| 2 | Saddened |
|  |  |
| **Quantity** | **Affect label** |
| 71 | Sadness |
| 1 | Scare |
| 50 | Scared |
| 1 | Scared Witle |
| 4 | Scary |
| 3 | Shame |
| 1 | Shameless |
| 1 | Shit |
| 5 | Shock |
| 5 | Shocked |
| 2 | Shocking |
| 5 | Sick |
| 5 | Sickened |
| 1 | Sickness |
| 1 | Slight Anger |
| 1 | Slight Anxiety |
| 1 | Slightly Scared |
| 1 | Solemn |
| 16 | Sorrow |
| 1 | Sorrow Awe |
| 1 | Sorrow Disgusting |
| 1 | Sorrow Fear |
| 1 | Sorrow Painful |
| 1 | Sorrow Pity |
| 1 | Sorrow Sad Fear |
| 1 | Sorrow Very |
| 8 | Sorrowful |
| 2 | Sorry |
| 1 | Spoil |
| 1 | Startled |
| 1 | Stressed |
| 2 | Suffering |
| 1 | Sulky |
| 1 | Superb |
| 6 | Surprise |
| 1 | Surprise By Gusteure |
| 2 | Surprised |
| 1 | Suspicios |
| 2 | Sympathetic |
| 18 | Sympathy |
| 1 | Sympathy sad |
| 1 | Tangled |
| 1 | Tearful |
|  |  |
| **Quantity** | **Affect label** |
| 3 | Tense |
| 1 | Terrible |
| 1 | Terrif |
| 5 | Terrific |
| 13 | Terrified |
| 2 | Terrifying |
| 8 | Terror |
| 3 | Threatened |
| 1 | Timid |
| 2 | Tired |
| 2 | Tormented |
| 2 | Trapped |
| 2 | Trepidation |
| 1 | Troubled |
| 3 | Uncertain |
| 1 | Uneasy |
| 1 | Unfortunate |
| 1 | Unhappiness |
| 9 | Unhappy |
| 1 | Unhappy About Future |
| 1 | Unhealthy |
|  |  |
|  |  |
| **Quantity** | **Affect label** |
| 1 | Uninterested |
| 1 | Unnerving |
| 1 | Unpleasant |
| 1 | Unsettled |
| 1 | Unwilling |
| 1 | Upsetting |
| 16 | Upset |
| 1 | Very Sad |
| 1 | Victimized |
| 1 | Violence Sad |
| 1 | Violence Sad Sorrow |
| 1 | Vomiting |
| 1 | Vulnerable |
| 1 | Wariness |
| 1 | Warm |
| 2 | Wary |
| 1 | Weak |
| 1 | Weary |
| 2 | Wonder |
| 1 | Wondering |
| 18 | Worried |
| 1 | Worrisome |
| 7 | Worry |

**III.c. Negative and Positive Affect Rating Distributions and Correlations**

**
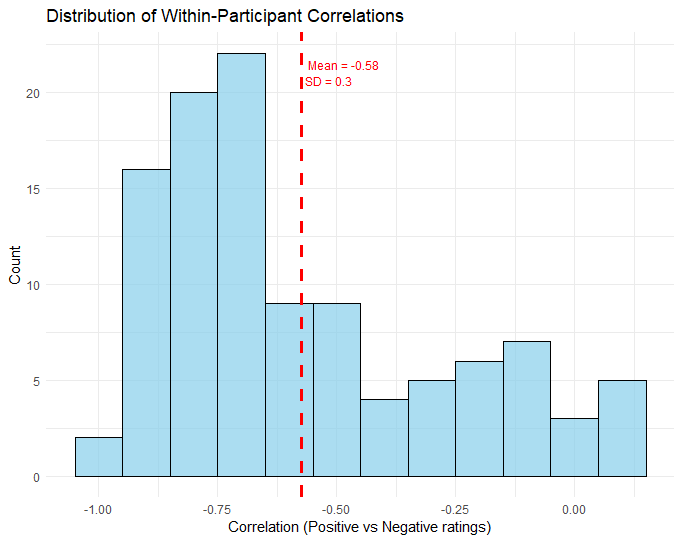
**
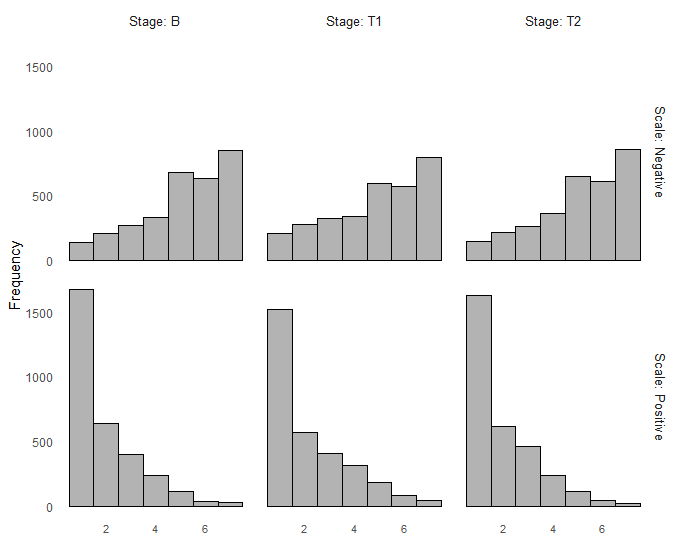


**III.d. Separate Analysis for the Negative Scale & the Positive Scale**

**Analysis of Negative Scale Data, using the data reported in the manuscript, i.e., without excluded participants.**

Descriptive Statistics per condition & Time point extracted from the model:

| Condition | Stage | Estimated Mean | SE | Lower CI | Upper CI |
| --- | --- | --- | --- | --- | --- |
| Look | Baseline | 5.14 | 0.245 | 4.66 | 5.62 |
|  | T1 | 5.22 | 0.258 | 4.72 | 5.73 |
|  | T2 | 5.14 | 0.260 | 4.63 | 5.65 |
| Name | Baseline | 5.29 | 0.260 | 4.78 | 5.79 |
|  | T1 | 5.47 | 0.279 | 4.92 | 6.01 |
|  | T2 | 5.39 | 0.281 | 4.84 | 5.94 |
| Reappraise | Baseline | 5.02 | 0.249 | 4.53 | 5.51 |
|  | T1 | 3.77 | 0.264 | 3.25 | 4.28 |
|  | T2 | 4.78 | 0.266 | 4.26 | 5.30 |
| Name  & Reappraise | Baseline | 4.76 | 0.247 | 4.27 | 5.24 |
|  | T1 | 4.69 | 0.261 | 4.17 | 5.20 |
|  | T2 | 4.87 | 0.263 | 4.35 | 5.39 |
| Confidence level used: 0.95 | | | | | |

|  | **df1** | **df2** | **F** | ***p*** | **η_p_^2^** |
| --- | --- | --- | --- | --- | --- |
| Reappraise | 1 | 106.97 | 10.72 | .001 | 0.09 |
| Name | 1 | 106.97 | 1.43 | .233 | 0.01 |
| Time | 2 | 99.44 | 8.25 | < .001 | 0.14 |
| Reappraise * Name | 1 | 106.97 | 0.01 | .918 | 0 |
| Reappraise * Time | 2 | 108.33 | 16.74 | < .001 | 0.24 |
| Name * Time | 2 | 108.33 | 10.58 | < .001 | 0.16 |
| Reappraise * Name *Time | 2 | 108.33 | 7.54 | < .001 | 0.12 |

| Note. Results are from Type III tests of fixed effects with Satterthwaite’s approximation for denominator degrees of freedom. |
| --- |


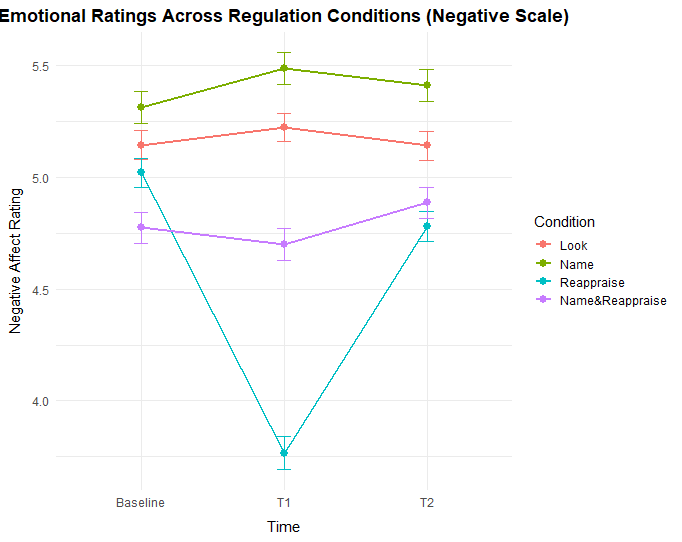


**Analysis of Positive Scale Data, using the data reported in the manuscript, i.e., without excluded particpants.**

Descriptive Statistics per condition & Time point extracted from the model:

| Condition | Stage | Estimated Mean | SE | Lower CI | Upper CI |
| --- | --- | --- | --- | --- | --- |
| Look | Baseline | 1.88 | 0.158 | 1.56 | 2.19 |
|  | T1 | 1.81 | 0.191 | 1.43 | 2.18 |
|  | T2 | 1.92 | 0.165 | 1.59 | 2.24 |
| Name | Baseline | 2.00 | 0.171 | 1.67 | 2.34 |
|  | T1 | 1.81 | 0.208 | 1.40 | 2.21 |
|  | T2 | 1.85 | 0.180 | 1.50 | 2.20 |
| Reappraise | Baseline | 1.91 | 0.162 | 1.59 | 2.23 |
|  | T1 | 3.30 | 0.196 | 2.92 | 3.68 |
|  | T2 | 2.29 | 0.196 | 1.96 | 2.62 |
| Name  & Reappraise | Baseline | 1.76 | 0.160 | 1.44 | 2.07 |
|  | T1 | 1.89 | 0.193 | 1.51 | 2.27 |
|  | T2 | 1.72 | 0.167 | 1.39 | 2.05 |
| Confidence level used: 0.95 | | | | | |

|  | **df1** | **df2** | **F** | ***p*** | **η_p_^2^** |
| --- | --- | --- | --- | --- | --- |
| Reappraise | 1 | 106.97 | 4.01 | .04 | 0.04 |
| Name | 1 | 106.97 | 6.60 | .01 | 0.06 |
| Time | 2 | 107.39 | 10.75 | < .001 | 0.17 |
| Reappraise * Name | 1 | 106.97 | 7.39 | .007 | 0.06 |
| Reappraise * Time | 2 | 108.18 | 21.69 | < .001 | 0.29 |
| Name * Time | 2 | 108.18 | 15.19 | < .001 | 0.22 |
| Reappraise * Name *Time | 2 | 108.18 | 8.60 | < .001 | 0.14 |

Note. Results are from Type III tests of fixed effects with Satterthwaite’s approximation for denominator degrees of freedom.


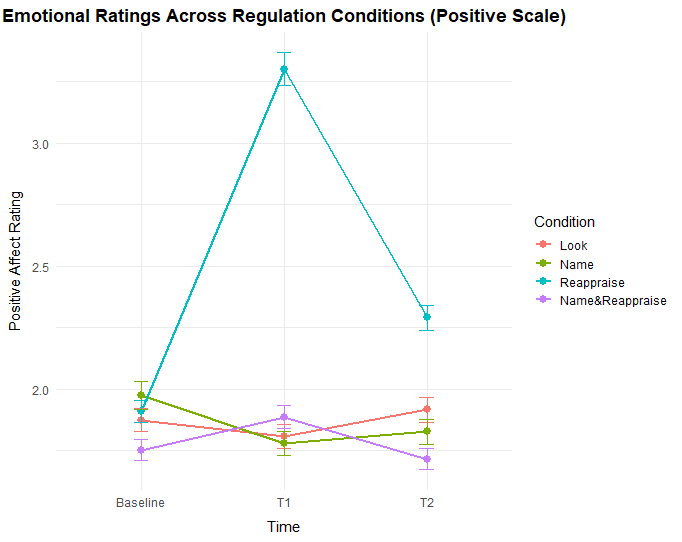


**III.e. Results with excluded participants**

Analysis including all the participants that were excluded due to judges' criteria. Participants that were excluded for not finishing either day of the study, or for beginning either session multiple times are not included in this analysis.

Descriptive Statistics per condition & Time point extracted from the model:

| Condition | Stage | Estimated Mean | SE | Lower CI | Upper CI |
| --- | --- | --- | --- | --- | --- |
| Look | Baseline | 5.67 | 0.183 | 5.31 | 6.03 |
|  | T1 | 5.75 | 0.205 | 5.53 | 6.15 |
|  | T2 | 5.66 | 0.188 | 5.29 | 6.02 |
| Name | Baseline | 5.70 | 0.186 | 5.34 | 6.07 |
|  | T1 | 5.89 | 0.209 | 5.48 | 6.30 |
|  | T2 | 5.84 | 0.192 | 5.46 | 6.21 |
| Reappraise | Baseline | 5.51 | 0.175 | 5.17 | 5.85 |
|  | T1 | 4.53 | 0.193 | 4.15 | 4.91 |
|  | T2 | 5.28 | 0.178 | 4.93 | 5.63 |
| Name  & Reappraise | Baseline | 5.53 | 0.178 | 5.18 | 5.88 |
|  | T1 | 5.48 | 0.198 | 5.09 | 5.87 |
|  | T2 | 5.59 | 0.182 | 5.24 | 5.95 |
| Confidence level used: 0.95 | | | | | |

|  | **df1** | **df2** | **F** | ***p*** | **η_p_^2^** |
| --- | --- | --- | --- | --- | --- |
| Reappraise | 1 | 129.03 | 11.71 | < .001 | 0.08 |
| Name | 1 | 129.03 | 4.78 | .02 | 0.04 |
| Time | 2 | 108.69 | 5.29 | .006 | 0.09 |
| Reappraise * Name | 1 | 129.03 | 1.54 | .216 | 0.01 |
| Reappraise * Time | 2 | 129.02 | 13.77 | < .001 | 0.18 |
| Name * Time | 2 | 129.02 | 9.92 | < .001 | 0.13 |
| Reappraise * Name *Time | 2 | 129.02 | 5.56 | < .001 | 0.08 |

| Note. Results are from Type III tests of fixed effects with Satterthwaite’s approximation for denominator degrees of freedom. |
| --- |

**
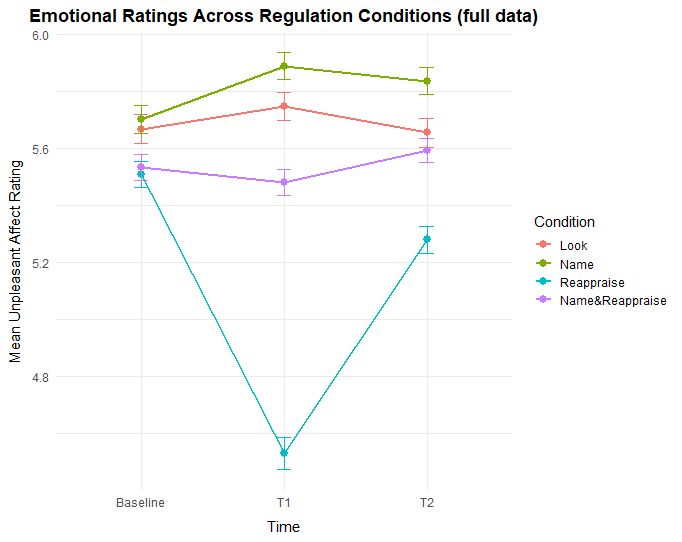
**

**VI. Study 2**

**VI.a. Participant Exclusion**

One-hundred and sixty-two participants began the study on the first day. Nine participants were excluded from data analysis for the following reasons:

- Two participants did not complete the task on the first day.
- Seven did not return to the second day (includes non-returners and non-finishers or duplicates eliminated from session 2).
- Two participants completed the task on the second day twice (some did not complete both attempts, but because stimuli were seen more than intended, these participants were excluded).

We ended up with N = 151 participants with eligible entries. The data of these participants was coded for valid reappraisal, valid affect labeling, and whether they understood the purpose of the study by 3 independent judges, which led to the exclusion of:

- Nineteen participants understood the purpose of the study.
- Sixteen participants failed to provide valid reappraisals (one of these was already excluded due to understanding the study purpose).
  - Six from the Name & Reappraise condition and 10 from the Reappraise condition
- One participants failed to provide valid affect labeling in more than a third of the trials (note that this participants also failed the reappraisal quality check).
- One participant rated all 24 images exactly the same on both the negative and the positive scales throughout a whole session, including the attention check images.

The final sample reported in the manuscript after all exclusions included N = 115 participants.

**VI.b. Affect labels Generated by Participants**

The table displays the original entries by participants; only undoubted spelling errors were corrected.

| **Quantity** | **Affect label** |
| --- | --- |
| 1 | Affection |
| 1 | Affectionate |
| 19 | Afraid |
| 1 | Afraid And Worried |
| 1 | Aggressive |
| 1 | Agony |
| 1 | Agravattion |
| 4 | Alarmed |
| 2 | Amazed |
| 1 | Amazement |
| 1 | Amused |
| 44 | Anger |
| 1 | Anger Isolation |
| 32 | Angry |
| 1 | Angry And So |
| 2 | Anguish |
| 5 | Annoyance |
| 1 | Annoye |
| 5 | Annoyed |
| 1 | Annoying |
| 1 | Anomosity |
| 1 | Anquish |
| 3 | Anxiety |
| 16 | Anxious |
| 1 | Appalled |
| 1 | Apprehensive |
| 2 | Ashamed |
| 1 | Astonished |
| 3 | Aversion |
| 15 | Awe |
| 1 | Awe Hes Cute |
| 1 | Awestruck |
| 3 | Awful |
| 3 | Bad |
| 1 | Beautiful |
| 1 | Bewildered |
| 2 | Bewilderment |
| 1 | Bitterness |
| 1 | Bleek |
| 1 | Blue |
| 1 | Bored |
| 1 | Boredom |
| **Quantity** | **Affect label** |
| 1 | Bright |
| 1 | Broken |
| 6 | Calm |
| 2 | Caring |
| 2 | Cautious |
| 1 | Chilled |
| 1 | Choke |
| 14 | Compassion |
| 3 | Compassionate |
| 19 | Concern |
| 10 | Concerned |
| 1 | Confuse |
| 14 | Confused |
| 12 | Confusion |
| 3 | Contempt |
| 1 | Cool |
| 1 | Courage |
| 1 | Crazy |
| 3 | Creepy |
| 1 | Crushed |
| 9 | Curiosity |
| 14 | Curious |
| 1 | Curious And Confused |
| 1 | Curious And Positive |
| 3 | Danger |
| 1 | Dangerous |
| 1 | Defensive |
| 1 | Dejected |
| 6 | Depressed |
| 3 | Despair |
| 5 | Devastated |
| 1 | Devastation |
| 1 | Devastation Depress |
| 3 | Dirty |
| 1 | Disagreement |
| 4 | Disappointed |
| 2 | Disappointment |
| 1 | Disater |
| 1 | Disbelief |
| 1 | Disconcern |
| 1 | Disdain |
| 3 | Disguised |
| **Quantity** | **Affect label** |
| 111 | Disgust |
| 1 | Disgust And Sadness |
| 1 | Disgustated |
| 58 | Disgusted |
| 1 | Disgusted And Nauseous |
| 1 | Disgusted And Saddened |
| 1 | Disgusted Grossed Out Stressed |
| 8 | Disgusting |
| 1 | Dishearten |
| 1 | Disheartened |
| 1 | Dislike |
| 1 | Dismay |
| 1 | Despairing |
| 1 | Despicable |
| 2 | Disappointed |
| 2 | Distressed |
| 3 | Disturbed |
| 1 | Disturbing |
| 1 | Drama |
| 4 | Dread |
| 2 | Dreadful |
| 1 | Dreary |
| 1 | Empathetic |
| 12 | Empathy |
| 1 | Emptiness |
| 2 | Empty |
| 1 | Energized |
| 1 | Enraged |
| 1 | Enthused |
| 1 | Excited Scared Curious Interested |
| 1 | Excitement |
| 1 | Extreme Sorrow |
| 1 | Fascination |
| 131 | Fear |
| 12 | Fearful |
| 1 | Fierce |
| 2 | Fine |
| 1 | Freaked Out |
| 2 | Fright |
| 2 | Frighten |
| 9 | Frightened |
| 1 | Frightning |
|  |  |
| **Quantity** | **Affect label** |
| 3 | Frustrated |
| 1 | Frustration |
| 4 | Funny |
| 1 | Furious |
| 1 | Furry |
| 1 | Gloom |
| 1 | Gloomy |
| 5 | Grief |
| 5 | Gross |
| 17 | Grossed Out |
| 2 | Guarded |
| 3 | Guilt |
| 1 | Guilty |
| 1 | Gutted |
| 1 | Gutting |
| 1 | Happiness |
| 5 | Happy |
| 2 | Hate |
| 1 | Hatred |
| 1 | Haunted |
| 5 | Heartbreak |
| 1 | Heartbreaking |
| 5 | Heartbroken |
| 1 | Heartless |
| 2 | Heartsick |
| 8 | Helpless |
| 1 | Helplessness |
| 4 | Hope |
| 5 | Hopeful |
| 3 | Hopeless |
| 1 | Hopelessness |
| 3 | Horrible |
| 16 | Horrified |
| 1 | Horrified And Distraught |
| 1 | Horrified And Sad |
| 1 | Horrified Disgusted And Scared |
| 1 | Horrified Disgusted Distraught Nauseous |
| 2 | Horrifying |
| 24 | Horror |
| 1 | Hostile |
| 7 | Hurt |
| 1 | Hurting |
|  |  |
| **Quantity** | **Affect label** |
| 1 | Ill |
| 1 | Impressed |
| 1 | In Danger |
| 2 | Indifference |
| 12 | Indifferent |
| 1 | Inhumane |
| 1 | Injustice |
| 13 | Interest |
| 9 | Interested |
| 1 | Interested Scared Curious |
| 4 | Intimidated |
| 3 | Intrigue |
| 3 | Intrigued |
| 1 | Irked |
| 4 | Irritated |
| 1 | Irritation |
| 1 | Isolation |
| 1 | Jumpy |
| 1 | Kindness |
| 1 | Laughing |
| 1 | Livid |
| 2 | Lonely |
| 1 | Longing |
| 1 | Loss |
| 1 | Lust |
| 8 | Mad |
| 1 | Mad And Concerned |
| 1 | Mad And Scared |
| 1 | Melancholic |
| 1 | Melancholy |
| 1 | Mercy And Angry |
| 1 | Miserable |
| 3 | Misery |
| 1 | Mixed Emotion Here |
| 1 | Morbid |
| 1 | Mortified |
| 2 | Mournful |
| 6 | Nasty |
| 1 | Nausea |
| 1 | Nauseated |
| 1 | Neglected |
| 8 | Nervous |
| 1 | Nervousness |
| 22 | Neutral |
| **Quantity** | **Affect label** |
| 5 | Normal |
| 1 | Nostalgia |
| 1 | Nostalgic |
| 1 | Nothing |
| 2 | Oppressed |
| 10 | Pain |
| 1 | Pained |
| 2 | Painful |
| 3 | Panic |
| 1 | Panicked |
| 1 | Peace |
| 1 | Perplexed |
| 1 | Pissed |
| 18 | Pity |
| 1 | Power |
| 3 | Protective |
| 1 | Puzzl |
| 2 | Puzzlement |
| 1 | Queasy |
| 2 | Rage |
| 1 | Regret |
| 2 | Regretful |
| 1 | Repelled |
| 1 | Repulsion |
| 1 | Resent |
| 2 | Resentful |
| 1 | Respect |
| 1 | Respectful |
| 1 | Revolted |
| 1 | Revolting |
| 2 | Revulsion |
| 208 | Sad |
| 1 | Sad And Angry |
| 1 | Sad And Nosta |
| 1 | Sad And Scared |
| 1 | Sad And Worried |
| 1 | Sad And Worried And Angry |
| 1 | Sad Horrified Scared Disgusted |
| 7 | Saddened |
| 1 | Sadn |
| 84 | Sadness |
| 2 | Scare |
| 88 | Scared |
| **Quantity** | **Affect** **label** |
| 1 | Scared And Worried |
| 7 | Scary |
| 1 | Serious |
| 3 | Shame |
| 1 | Shameful |
| 9 | Shock |
| 15 | Shocked |
| 1 | Shocking |
| 4 | Sick |
| 2 | Sick To Stomach |
| 1 | Sickened |
| 1 | Slight Fear |
| 1 | So Sad |
| 18 | Sorrow |
| 2 | Sorrowful |
| 4 | Sorry |
| 1 | Sorry And Pity |
| 1 | Speechless |
| 1 | Spooked |
| 1 | Startled |
| 1 | Strange |
| 3 | Stressed |
| 1 | Stuck |
| 10 | Surprise |
| 6 | Surprised |
| 2 | Suspense |
| 3 | Sympathetic |
| 8 | Sympathy |
| 3 | Tension |
|  |  |
|  |  |
| **Quantity** | **Affect** **label** |
| 3 | Terrible |
| 15 | Terrified |
| 2 | Terrifying |
| 3 | Terror |
| 1 | Threat |
| 1 | Threatened |
| 1 | Tragic |
| 2 | Trapped |
| 2 | Traumatized |
| 1 | Unbelief |
| 3 | Uncomfortable |
| 1 | Undecided |
| 6 | Uneasy |
| 1 | Unjust |
| 2 | Unsettled |
| 1 | Unsure |
| 9 | Upset |
| 3 | Very Sad |
| 1 | Very Sad And |
| 1 | Very Upset |
| 3 | Weird |
| 6 | Wonder |
| 13 | Worried |
| 1 | Worried And Concerned |
| 2 | Worried And Sad |
| 4 | Worried And Scared |
| 1 | Worried And Stressed |
| 1 | Worried Scared Sad |
| 10 | Worry |
| 1 | Worrying |
| 1 | Yuck |

**VI.c. Negative and Positive Affect Rating Distributions and Correlations**


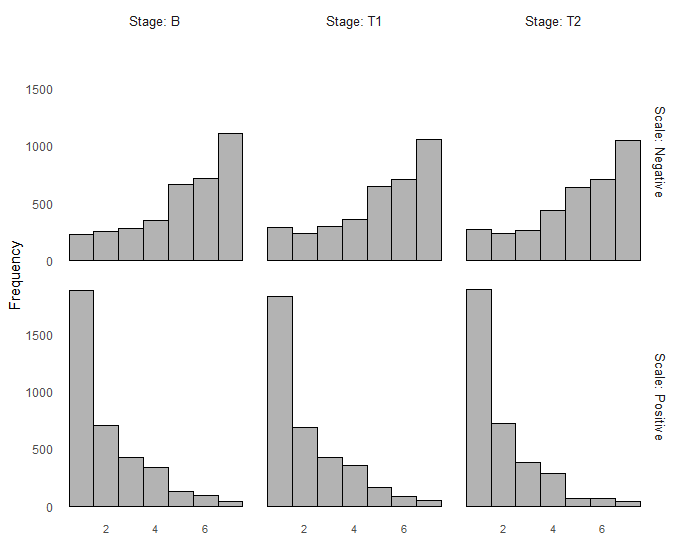


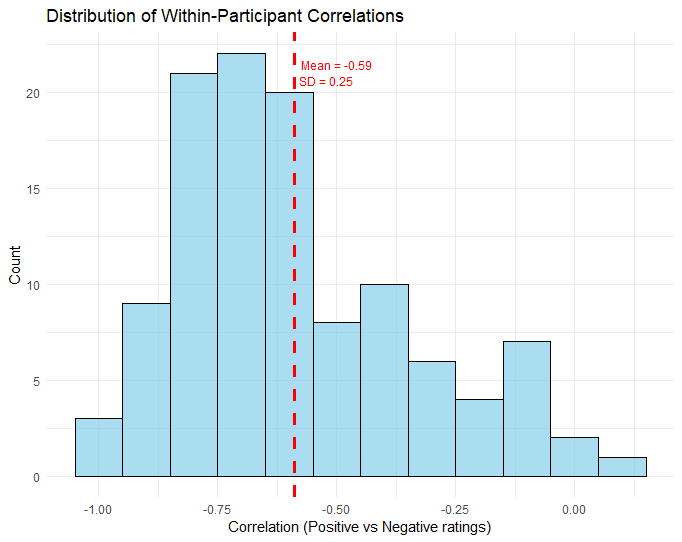


**III.d. Separate Analysis for the Negative Scale & the Positive Scale**

**Analysis of Negative Scale Data, using the data reported in the manuscript, i.e., without excluded participants.**

Descriptive Statistics per condition & Time point extracted from the model:

| Condition | Stage | Estimated Mean | SE | Lower CI | Upper CI |
| --- | --- | --- | --- | --- | --- |
| Look | Baseline | 4.92 | 0.249 | 4.43 | 5.40 |
|  | T1 | 5.01 | 0.254 | 4.51 | 5.51 |
|  | T2 | 4.93 | 0.261 | 4.42 | 5.44 |
| Name | Baseline | 5.08 | 0.249 | 4.60 | 5.57 |
|  | T1 | 5.28 | 0.254 | 4.78 | 5.78 |
|  | T2 | 5.09 | 0.262 | 4.58 | 5.60 |
| Reappraise | Baseline | 5.15 | 0.293 | 4.57 | 5.72 |
|  | T1 | 4.37 | 0.309 | 3.76 | 4.98 |
|  | T2 | 4.92 | 0.318 | 4.29 | 5.54 |
| Name  & Reappraise | Baseline | 5.19 | 0.280 | 4.64 | 5.74 |
|  | T1 | 5.30 | 0.294 | 4.72 | 5.87 |
|  | T2 | 5.01 | 0.301 | 4.42 | 5.60 |
| Confidence level used: 0.95 | | | | | |

|  | **df1** | **df2** | **F** | ***p*** | **η_p_^2^** |
| --- | --- | --- | --- | --- | --- |
| Reappraise | 1 | 111 | 0.07 | .784 | 0 |
| Name | 1 | 111 | 1.51 | .221 | 0.01 |
| Time | 2 | 100.34 | 1.46 | .236 | 0.03 |
| Reappraise * Name | 1 | 111 | 0.11 | .730 | 0 |
| Reappraise * Time | 2 | 110.93 | 6.94 | .001 | 0.11 |
| Name * Time | 2 | 110.93 | 8.82 | < .001 | 0.14 |
| Reappraise * Name *Time | 2 | 110.93 | 5.29 | .006 | 0.09 |

| Note. Results are from Type III tests of fixed effects with Satterthwaite’s approximation for denominator degrees of freedom. |
| --- |


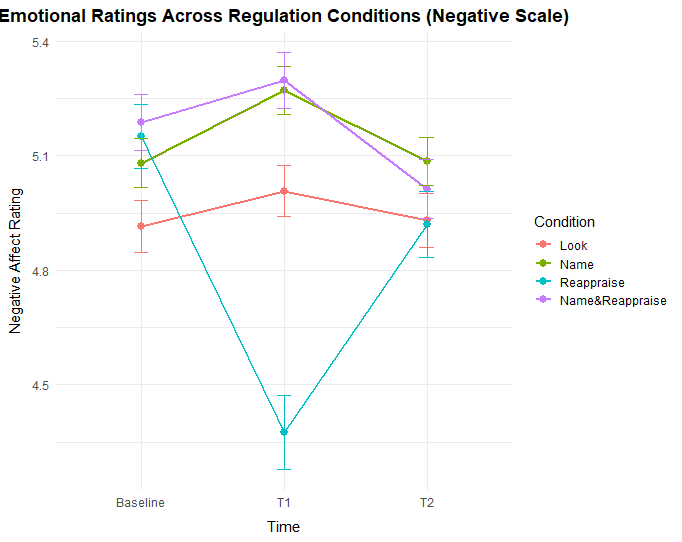


**Analysis of Positive Scale Data, using the data reported in the manuscript, i.e., without excluded participants.**

Descriptive Statistics per condition & Time point extracted from the model:

| Condition | Stage | Estimated Mean | SE | Lower CI | Upper CI |
| --- | --- | --- | --- | --- | --- |
| Look | Baseline | 2.15 | 0.163 | 1.83 | 2.46 |
|  | T1 | 2.11 | 0.162 | 1.79 | 2.43 |
|  | T2 | 2.17 | 0.158 | 1.86 | 2.48 |
| Name | Baseline | 1.99 | 0.163 | 1.67 | 2.30 |
|  | T1 | 1.80 | 0.162 | 1.49 | 2.12 |
|  | T2 | 1.74 | 0.158 | 1.43 | 2.05 |
| Reappraise | Baseline | 2.21 | 0.194 | 1.83 | 2.59 |
|  | T1 | 2.81 | 0.196 | 2.42 | 3.19 |
|  | T2 | 2.23 | 0.191 | 1.86 | 2.60 |
| Name  & Reappraise | Baseline | 1.79 | 0.185 | 1.43 | 2.16 |
|  | T1 | 1.74 | 0.186 | 1.38 | 2.11 |
|  | T2 | 1.71 | 0.182 | 1.36 | 2.07 |
| Confidence level used: 0.95 | | | | | |

|  | **df1** | **df2** | **F** | ***p*** | **η_p_^2^** |
| --- | --- | --- | --- | --- | --- |
| Reappraise | 1 | 111.07 | 0.45 | .5 | 0 |
| Name | 1 | 111.06 | 12.51 | < .001 | 0.10 |
| Time | 2 | 103.75 | 4.03 | .02 | 0.07 |
| Reappraise * Name | 1 | 111.06 | 1.82 | .179 | 0.02 |
| Reappraise * Time | 2 | 111.08 | 5.56 | .004 | 0.09 |
| Name * Time | 2 | 111.08 | 6.16 | .002 | 0.10 |
| Reappraise * Name *Time | 2 | 111.08 | 4.41 | .014 | 0.07 |

| Note. Results are from Type III tests of fixed effects with Satterthwaite’s approximation for denominator degrees of freedom. |
| --- |


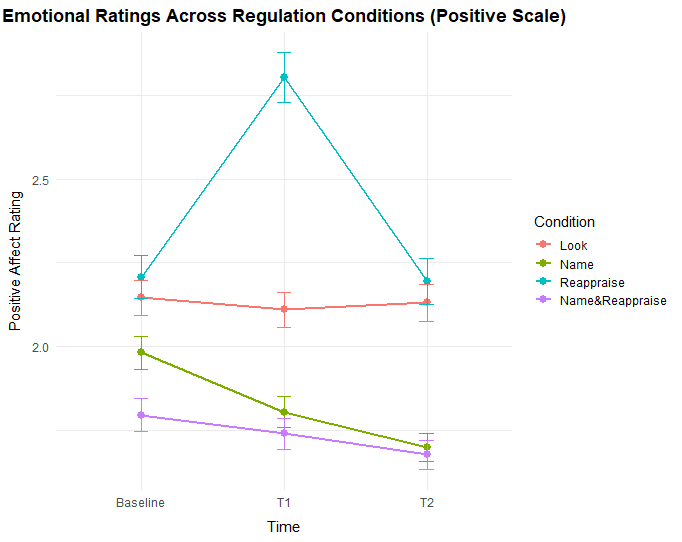


**VI.e. Results with excluded participants**

Analysis including all the participants that were excluded due to judges' criteria. Participants that were excluded for not finishing either day of the study, for beginning either session multiple times, or for answering a single value to all images (including attention checks), are not included in this analysis.

Descriptive Statistics per condition & Time point extracted from the model:

| Condition | Stage | Estimated Mean | SE | Lower CI | Upper CI |
| --- | --- | --- | --- | --- | --- |
| Look | Baseline | 5.43 | 0.181 | 5.08 | 5.79 |
|  | T1 | 5.47 | 0.177 | 5.12 | 5.82 |
|  | T2 | 5.40 | 0.185 | 5.04 | 5.76 |
| Name | Baseline | 5.55 | 0.180 | 5.19 | 5.90 |
|  | T1 | 5.74 | 0.176 | 5.39 | 6.08 |
|  | T2 | 5.66 | 0.184 | 5.30 | 6.02 |
| Reappraise | Baseline | 5.45 | 0.180 | 5.10 | 5.80 |
|  | T1 | 4.78 | 0.176 | 4.44 | 5.13 |
|  | T2 | 5.32 | 0.184 | 4.96 | 5.68 |
| Name  & Reappraise | Baseline | 5.67 | 0.181 | 5.32 | 6.03 |
|  | T1 | 5.4879 | 0.177 | 5.44 | 6.14 |
|  | T2 | 5.64 | 0.185 | 5.28 | 6.00 |
| Confidence level used: 0.95 | | | | | |

|  | **df1** | **df2** | **F** | ***p*** | **η_p_^2^** |
| --- | --- | --- | --- | --- | --- |
| Reappraise | 1 | 145.98 | 0.63 | .426 | 0 |
| Name | 1 | 145.98 | 8.81 | .003 | 0.06 |
| Time | 2 | 129.91 | 1.05 | .351 | 0.02 |
| Reappraise * Name | 1 | 145.98 | 1.52 | .219 | 0.01 |
| Reappraise * Time | 2 | 146.04 | 7.02 | .001 | 0.09 |
| Name * Time | 2 | 146.04 | 9.93 | < .001 | 0.12 |
| Reappraise * Name *Time | 2 | 146.04 | 5.85 | .003 | 0.07 |

| Note. Results are from Type III tests of fixed effects with Satterthwaite’s approximation for denominator degrees of freedom. |
| --- |


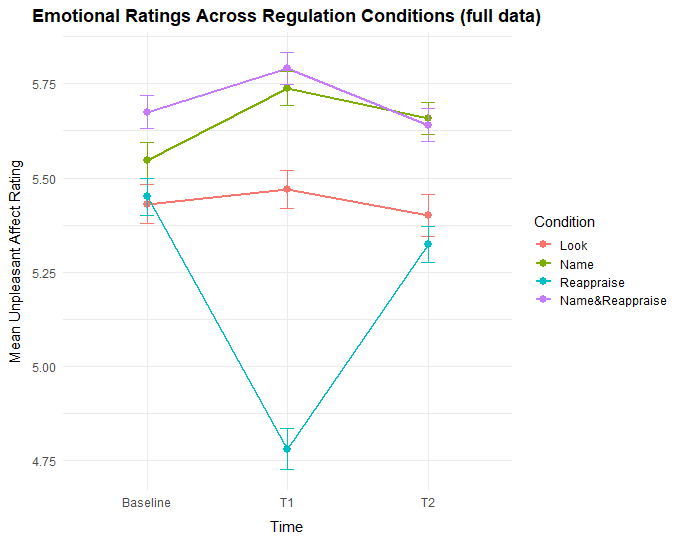

Supplement: Supplementary file 1 — Supplementary Material 1 (DOCX 183 KB) [file 42761_2026_362_MOESM1_ESM.docx]
